# Accelerating an integrative view of quantum biology

**DOI:** 10.3389/fphys.2023.1349013

**Published:** 2024-01-11

**Authors:** Luke J. Kinsey, Wendy S. Beane, Kelly Ai-Sun Tseng

**Affiliations:** ^1^ Biology Department, Kalamazoo College, Kalamazoo, MI, United States; ^2^ Department of Biological Sciences, Western Michigan University, Kalamazoo, MI, United States; ^3^ School of Life Sciences, University of Nevada, Las Vegas, Las Vegas, NV, United States

**Keywords:** quantum biology, quantum technology, quantum physics, spin chemistry, non-trivial quantum effects

## Abstract

Quantum biology studies span multiple disciplines including physics, engineering, and biology with the goal of understanding the quantum underpinnings of living systems. Recent findings have brought wide attention to the role of quantum mechanisms in the function and regulation of biological processes. Moreover, a number of activities have been integral in building a vibrant quantum biology community. Due to the inherent interdisciplinary nature of the field, it is a challenge for quantum biology researchers to integrate and advance findings across the physical and biological disciplines. Here we outline achievable approaches to developing a shared platform—including the establishment of standardized manipulation tools and sensors, and a common scientific lexicon. Building a shared community framework is also crucial for fostering robust interdisciplinary collaborations, enhancing knowledge sharing, and diversifying participation in quantum biology. A unified approach promises not only to deepen our understanding of biological systems at a quantum level but also to accelerate the frontiers of medical and technological innovations.

## 1 Introduction: the emerging field of quantum biology

Quantum biology has emerged as a space for true scientific discourse, where cross-disciplinary research has built and is continuing to improve upon a scientific foundation that promises to drive the scientific revolutions of the past century into a new era of discovery. To capitalize on the successes of modern interdisciplinary fields like biophysics, the multiple fields in quantum biology have converged to become a global community of scientists who seek to identify the quantum underpinnings of life. The foundations of quantum biology can easily be seen in the literature, where research by physicists, biologists, chemists, and engineers has converged on overlapping interests such as: photosynthesis and catalysts (particle/energy transfer), magnetoreception, non-chemical signaling (light-based), metabolism (mitochondrial respiration), imaging and olfaction (sensing) (Blomberg and Siegbahn, 2010; Marais et al., 2018; Wang et al., 2022; Mould et al., 2023).

The extant literature exemplifies the rich dividends that are being yielded from such cross-disciplinary ventures. Data reveal that the ability of birds to migrate across great distances is underpinned by magnetically sensitive cryptochrome proteins in the avian eye used to detect the Earth’s geomagnetic field, where radical pair formation is thought to induce signaling that acts as a biological compass (Ritz et al., 2000; Xu et al., 2021; Tonelli et al., 2023). Interestingly other migratory animals, including fish, can also detect magnetic fields to navigate their environment (Naisbett-Jones and Lohmann, 2022). Studies have also shown that during photosynthesis the transfer of electrons and dissipation events are governed by quantum mechanical behaviors (Cao et al., 2020). In addition, mounting evidence suggests that the functions of biological catalysts (enzymes) are a result of quantum phenomena like tunneling (Brookes, 2017). Emerging research in cancer therapeutics and regenerative medicine continues to chart the intersection of quantum technologies with living systems, where current data demonstrate that exposure to magnetic fields predictably alter tissue growth in cancer cell culture and during regeneration events *in vivo* (Gurhan et al., 2021; Kinsey et al., 2023).

These advances are currently heralding in a new period of scientific investigation. Our present genomic era has benefited from repeated innovations that have become part of our fundamental understanding and methodology. For example, half a century ago biological focus was at the level of individual gene function. This was followed by the study of signaling pathway cascades, which led to interest in pathway integration via gene regulatory networks. The result led to today’s comparative analyses of entire genomes that permeate all aspects of scientific research. The accumulation of knowledge at each of these stages helped to push the scientific community into revolutionary new spaces (Kuhn, 1962). Quantum biology has the potential to become the next critical paradigm shift, with implications for basic cell biology, therapeutics, and cutting-edge technology.

## 2 Moving towards commonality

The community has begun to recognize that certain criteria and metrics will first need to be attained in order to facilitate a quantum biology revolution. The unique nature of this emerging field (which integrates aspects of biology, physics, chemistry, engineering, and clinical science) necessitates a common framework to ensure coherence and comparability across diverse research endeavors. The gap lies in the disparate methodologies and tools employed across labs and disciplines. This hinders the field’s ability to construct a cohesive narrative, and thus makes it more difficult to compare and integrate findings from various research groups.

Our perspective is that one key to bridging this gap will be the establishment of standardized practices in three primary categories. (1) Manipulation: the tools used to cause effects. (2) Sensors: the tools used to perform measurements. And (3) Vocabulary: the way in which results, gaps, and insights are communicated. By more precisely defining our manipulation tools, sensors, and terminology we remove large constraints of access (fewer information silos) and promote innovation (more collaborations).

### 2.1 Manipulation

Manipulation tools are instrumental to create specific conditions or stimuli to observe quantum effects in living systems (or classical outcomes altered by quantum phenomena). Currently, the field has a plethora of manipulation tools, which by and large are all custom built. This leads to differences in experimental design and hampers the ability to compare results across data sets. For instance, the approach to creating magnetic fields in experiments varies significantly among labs and within disciplines. This diversity can result in confounding variables that affect the ability to replicate and relate results across different domains. To advance the field, the development of standardized manipulation tools will provide a common platform for conducting experiments. This will also facilitate collaboration and enhance the ability of researchers to share current knowledge. These standards would allow for wider incorporation of results across the community and increase the possibilities for experiments across different model systems, allowing for the field to expand faster.

### 2.2 Sensors

Sensors are required for the measurement of quantum phenomena in biological systems, which is a critical step in connecting theory to the biology. The precision and sensitivity for such measurements demand that sensors be consistent to ensure data accuracy and comparability. Currently, labs often use custom-built or adapted sensors, which magnifies variations in data collection methods and outputs. Developing standardized sensors allows the community to ensure reliable and comparable data across different systems, leading to a more comprehensive understanding of quantum effects in biology. It is also worth considering when designing uniform sensors for quantum biology how these measurements will be incorporated into the data from classical studies of biological processes. This integration at the assay level will require collaborative efforts at the initial design stage. Related to this, concerted efforts to agree on the common critical measurements essential to the field will support building sensors that have wide applicability across the community.

### 2.3 Vocabulary

The quantum biology field is characterized using diverse vocabularies and reporting styles, reflective of its many constituents. This linguistic barrier is multi-fold: not only do the disparate quantum biology disciplines use different jargon and terminology, there is an assumed understanding of basic science concepts specific to each sub-field that is not comparable between disciplines. For example, the average biological researcher is not normally conversant with Pauli’s exclusion principle or the Zeeman effect, while the typical physicist has not studied cis-regulatory elements or signaling pathway dynamics. The standardization of the way we represent and report data (results), identify unknowns and issues (gaps), and interpret data (insights) would expedite collaboration. Development of a unified vocabulary and consistent reporting guidelines will facilitate clearer and more effective communication among researchers from various backgrounds. In addition, the establishment of a common vernacular and nomenclature would increase public understanding of quantum biology’s advances and aims.

## 3 Ongoing efforts

A number of organizations and groups have laid the groundwork to build consensus in the quantum biology field. The first steps have been focused on community building, bringing together researchers from diverse backgrounds to share insights and discuss a common path. These efforts include the Leverhulme Quantum Biology Doctoral Training Centre (QB-DTC) at the University of Surrey, the California NanoSystems Institute’s (CNSI) Quantum Biology Center at UCLA, and a Quantum Biology Research Coordination Network grant funded by the National Science Foundation. This network has helped to establish core community events like the Big Quantum Biology Meetings, a weekly global online seminar series hosted by QB-DTC and the Quantum Biology Tech (QuBiT) Lab at UCLA.

In March 2023, the newly established Quantum Biology Gordon Research Conference (QB-GRC) was held. As a component of this event, a week-long virtual Quantum Biology Bootcamp was offered. This free event introduced trainees and researchers in multiple disciplines to basic quantum biology concepts across both biology and physics. This represented an initial first step towards addressing the need for a common vocabulary and core cross-disciplinary concepts. The second QB-GRC is scheduled for 2025 and will be preceded by the inaugural Quantum Biology Gordon Research Seminar (QB-GRS). The QB-GRS provides trainees (students and postdoctoral fellows) the platform to network and interact across disciplines and facilitates the establishment of a shared language.

Across all these events is a focus on expanding participation in quantum biology research. An example is the Quantum Biology Interdisciplinary Trainee Exchange (QBite) program at Western Michigan University, which provides cross-disciplinary visiting scientist opportunities for trainees interested in joining the field. Shared experiences like these will promote the discussions needed to create common tools and measurements. Standardization efforts will also increase buy-in from a diverse group of established researchers, as well as facilitate efforts to promote interest in quantum biology from the next-generation.

## 4 Discussion: towards a collaborative future

As summarized above, current efforts have included a large focus on the development of a common vocabulary. While continued efforts in this direction will be needed—for example, a textbook on quantum biology, significant strides in community building have been made. With this strong foundation, now is the opportune time to leverage this community to focus on the other two areas (manipulation and sensors) as quantum biology enters its next phase of evolution. For manipulation, possible mechanisms to promote common methodologies would be to increase accessibility to current tools by publishing protocols and/or moving tool production to the commercial sphere. Developing a consistent set of sensors will likely take more effort to resolve, as it has the most complexity. This includes the diversity of model systems studied and the wide range of possible measurement tools.

To move forward, it is crucial we remember that standardization is not about rigid conformity but about the creation of a dynamic framework that can evolve alongside the field. It is about setting a common language and toolkit that can adapt to the rapidly evolving nature of quantum biology. A standardized framework also promises to democratize and diversify participation in this field. In order to decrease the number of barriers to entry and make the field more accessible, we must invite contributions from a broader range of scientists, particularly underrepresented groups. This inclusivity not only enriches the field with a variety of perspectives but also fosters a more collaborative and animated scientific community. The integration of numerous disciplines and the cross-pollination of ideas are fundamental to this field; standardization serves as a catalyst for this integration.

The promise of quantum biology, from revolutionizing cancer therapeutics to advancing our understanding of complex biological systems, can be significantly amplified through a coordinated approach. The field now possesses an established and vibrant global community. The path forward requires a concerted effort from all stakeholders. As the field continues to expand, standardization of tools, methods, and communication will not only increase the impact of the field but also pave the way for a more inclusive and collaborative research environment, accelerating discovery and innovation.

## Data Availability

The original contributions presented in the study are included in the article/Supplementary material, further inquiries can be directed to the corresponding authors.
